# Efficacy of Artificial Intelligence–Assisted Appliances in the Selection of Tooth Shade: Protocol for an Observational Study

**DOI:** 10.2196/68160

**Published:** 2025-12-22

**Authors:** Namita Zilpilwar, Sharayu Nimonkar, Surekha Godbole, Vikram Belkhode

**Affiliations:** 1 Department of Prosthodontics Crown and Bridge Sharad Pawar Dental College and Hospital Datta Meghe Institute Health Education and Research Wardha, Maharashtra India

**Keywords:** intraoral scanner, vita classic, CEILAB coordinates, smartphone, DSLR camera

## Abstract

**Background:**

Accurate shade matching in dentistry is crucial for achieving aesthetic outcomes, with increasing patient expectations driving advancements in shade selection technologies. Color perception is influenced by multiple factors such as incident light, reflection, absorption, observer variability, and environmental conditions. The evolution of shade-matching tools now includes digital and artificial intelligence (Al)-assisted appliances aimed at improving accuracy and ease of use.

**Objective:**

This study aims to compare and evaluate the efficacy of AI-assisted appliances, namely, smartphone cameras, digital single-lens reflex (DSLR) cameras, and intraoral scanners in selecting tooth shades in clinical practice.

**Methods:**

This observational study conducted at the Department of Prosthodontics and Crown & Bridge aims to evaluate shade selection methods in 221 participants recruited from the outpatient department based on specific inclusion and exclusion criteria, including age, oral health status, and informed consent. Three devices will be used for shade selection: a smartphone camera (iPhone 12, iOS 12.5.2) for quick, noninvasive, and accessible image capture; a DSLR camera (Canon EOS 200D) to obtain high-resolution images under standardized lighting for enhanced color accuracy; and an intraoral scanner (CEREC Primescan, Dentsply Sirona) offering precise 3D mapping and digital shade analysis. This multidevice approach allows for a comparative evaluation of conventional and advanced digital tools in clinical shade matching. The primary objective is to assess the effectiveness of commonly available digital teeth in accurately selecting tooth shades. Our study anticipates the following outcomes: validation of smartphone cameras as simple, economical, and efficient tools for basic shade matching; demonstration of DSLR cameras’ superiority in resolution and lighting control for improved accuracy; and confirmation of intraoral scanners as precise, customizable devices that offer a high level of digital integration. Statistical analyses will include sensitivity, specificity, and subgroup evaluations to compare the performance of each device. The findings are expected to show that both DSLR and smartphone cameras can match the effectiveness of intraoral scanners, offering viable alternatives for clinical use.

**Results:**

This study was intramurally funded in December 2024. Data collection is scheduled to commence following the publication of this study protocol. As of submission, no participants have been recruited, and data analysis is yet to begin. Results are expected to be completed and published in early January 2026.

**Conclusions:**

This study aims to establish a standard protocol for the use of Al-assisted, easily accessible tools such as smartphones and DSLRs for dental shade selection. These devices, being user-friendly and nontechnical, could democratize the process of shade matching, benefiting both clinicians and patients by improving restoration outcomes while reducing costs and complexity. Our results will contribute to the growing body of digital dentistry literature and support the integration of practical Al tools in everyday clinical practice.

**Trial Registration:**

Clinical Trials Registry-India CTRI/2024/07/070002; https://tinyurl.com/4pt5eutb

**International Registered Report Identifier (IRRID):**

PRR1-10.2196/68160

## Introduction

Aesthetic dentistry has seen a vast demand to meet the requirements of the patients to achieve a perfect smile and is playing a vital role in contemporary dentistry. To ultimately meet the aesthetic requirements, dental restorations must precisely mimic the shade of the teeth [1]. The instruments used for shade matching are constantly evolving in dentistry due to the growing demand from patients for more aesthetic results. Numerous factors can affect the intricate process of color perception, including incident light, light reflection, light absorption, the observer's visual matching, and the object's surroundings [2].

Shade selection is broadly classified into 2 methods, namely, subjective and objective methods. Subjective methods rely on traditional tools such as the conventional Vita Shade Guide System and Vita Classic Shade Guide, while objective methods use advanced instruments, often powered by artificial intelligence (AI), including spectrophotometers, colorimeters, and intraoral scanners [3]. The accuracy of the subjective approaches used to choose shades for the vita shade guide and vita classical shade guide is influenced by the chromatic perception disability. Numerous factors such as the environment of the clinics and the reflection of light contribute to subjective techniques [4].

AI-based systems minimize human error in tooth shade selection, which is often affected by subjective factors such as lighting conditions, visual fatigue, and individual perception [5]. AI algorithms analyze digital images of teeth in real time, ensuring a more consistent and accurate shade selection by comparing them to vast databases of tooth shades. AI technology can be integrated with intraoral scanners, spectrophotometers, and colorimeters to automatically identify and suggest the most accurate shade. AI refines measurements by eliminating inconsistencies, compensating for lighting variations, and ensuring that the most accurate shade is selected based on the collected data [3].

A spectrophotometer is considered the gold standard for this purpose [6]. The spectrophotometer utilizes a 6500K light source to accurately match tooth color and displays the results by using a visual shade guide. Despite certain drawbacks such as the need for costly, nonintuitive, and technique-sensitive equipment, spectrophotometry remains a versatile and highly efficient method for shade matching [6]. However, given its limitations such as cost and limited accessibility in small dental practices, portable alternatives such as colorimeters were introduced to provide a more practical option for shade matching [6].

A colorimeter, which measures an object's spectrum reflectance or transmittance to determine its color attributes, is an alternative for choosing digital shades [7]. They usually use precise light sources and sensors to measure tooth color accurately. To determine how much light is reflected or transmitted onto the tooth, the colorimeter emits light onto it. Utilizing this measurement, the gadget provides numerical values in a given color space, like CIELAB coordinates [8]. However, this technique's drawback is that it may not be as exact at reproducing hues, especially for teeth with complex tones or translucency. Additionally, environmental factors such as illumination can affect colorimeter readings, requiring exact calibration and standardization [8].

Recent advancements in dentistry have led to an increase in the use of intraoral scanners, some of which can produce vivid images and distinguish between soft and hard tissues. Shade color information from intraoral scanners is also based on the visual shade criteria [9]. Additionally, scanners are able to identify tooth color and distinguish between the architecture of teeth and the oral cavity. The cost of the technology is one of the most significant challenges, and only some smaller or wealthier clinics may be able to afford it. The use of digital single-lens reflex (DSLR) and smartphone pictures as AI tools for choosing tooth shades is becoming more popular in modern dentistry as a solution to these problems because of their capacity to integrate sophisticated AI-driven analysis with excellent image capturing. Finding the ideal shade for dental restorations is made easier, more accessible, and more precise by these technologies. DSLR cameras provide high resolution and excellent image quality, making it possible to see the tooth's surface, texture, and translucency in great detail. AI software can subsequently process these photos to more precisely determine the shade. The number of dentists using their smartphones for photography is rapidly increasing due to the complex operation and manipulation of DSLR cameras [10].

However, the accuracy of evaluating the tooth shade by using smartphone cameras may vary based on a number of factors such as lighting and camera quality. The dentist can enhance the image for better shade-matching results by adjusting the camera angle or lighting conditions based on real-time feedback from AI algorithms on smartphones. Nonetheless, it remains a feasible and reachable substitute [11]. In this study, we will compare the efficacy of DSLR and smartphone cameras against that of intraoral scanners for selecting tooth shades. In order to accomplish this, factors such as color accuracy, image quality, and clinical user-friendliness will be assessed. This kind of comparison could help to determine the most effective method for accurately determining the tooth shade in dentistry. Posterior teeth will be included in this study, as they will be included as additional variables to assess the potential differences in shade selection accuracy across tooth types. This may influence the outcomes due to variations in morphology, translucency, and light reflection. Thus, the objective of this study is to evaluate the scope of readily accessible AI-assisted appliances for determining dental shades in aesthetic dentistry.

## Methods

### Study Design and Participants

This will be an observational study conducted in the Department of Prosthodontics and Crown & Bridge at Sharad Pawar Dental College and Hospital, Sawangi, Wardha, Maharashtra, India. The inclusion criteria and exclusion criteria are shown in Textbox 1. To reduce observer bias, the individuals responsible for analyzing and comparing the shade-matching outcomes (based on laboratory values) will be blinded to the device used for capturing the tooth shade (smartphone, DSLR, or intraoral scanner). Each image or scan will be coded and deidentified prior to analysis, ensuring that the evaluators are unaware of the imaging modality. All data will be stored securely and entered into a centralized, password-protected database. Double data entry will be employed for cross-verification, and all files will be time-stamped and logged for traceability.

**Inclusion and exclusion criteria for this study.**

**Inclusion criteria**

- Co-operative patients who are not anxious and are not apprehensive
- Anterior maxillary central incisors will be used
- Patients willing to participate in the study

**Exclusion criteria**

- Teeth with any abrasion
- Teeth with enamel anomalies
- Adjacent teeth with crown prosthesis
- Teeth with calculus and stains (extrinsic and intrinsic)
- Root canal–treated teeth
- Teeth with restorations
- Fractured tooth (vertical and horizontal)

### Data Collection Procedure

A total of 221 participants will be recruited from the outpatient department. To choose their shade, study participants will be exposed to an intraoral scanner, a smartphone, and a DSLR. The study participants will be informed about the study and asked to sign a consent form prior to the procedure. The participants will be instructed to clean their teeth and take off any makeup before the test. The treatment will be performed on the patients' maxillary central incisors by using 3 different technologies: an intraoral scanner, a DSLR, and a smartphone camera.

Shade selection using a smartphone (iPhone 12; software version iOS 12.5.2.) will be performed using natural daylight or daylight-mimicking LED lights that are ideal for accurate color reproduction. The smartphone camera will be positioned parallel to the patient's face at a distance of 30 cm and zoomed in to focus on the teeth of interest, that is, the maxillary central incisors. Photos will be then imported into the Adobe Photoshop software to obtain the L*a*b values.

Photographic color selection will be done using the DSLR camera (Canon EOS 200D) with a macro lens for close-up shots with high detail and resolution. The DSLR camera will be set to manual mode to grant full control over exposure, aperture, and white balance settings, allowing for optimal customization to capture high-quality images for shade selection. The tooth would be placed at a distance of about 22-28 centimeters from the DSLR camera. The L*a*b values will then be acquired by importing the photographs into the Adobe Photoshop software.

For color selection, the Cerec Primescan (Dentsply Sirona) intraoral scanner will be used. The vita classic color selection mode will be activated in the scanner's software. We will scan the maxillary central incisor and note the color that is detected. The L*a*b values will be determined.

### Ethical Considerations

This study underwent ethical review and approval by the Datta Meghe Institute of Higher Education and Research institutional review board as per institutional guidelines (approval DMIHER[DU]IEC/2024/241). Informed consent will be obtained from all the participants involved in the study. The participants will be clearly informed about the study's purpose, procedures, potential risks, and benefits, specifically regarding the use of AI-assisted devices for tooth shade selection. Participant data will be anonymized to ensure privacy and confidentiality. Identifying information will not be included in any reports or publications. Strict data security measures, including encryption and secure storage, will be implemented to protect all participant information. Participants will be compensated for their time and involvement in the study with a modest stipend or gift card, the value of which will be specified in the informed consent form. Compensation will be fair and consistent for all participants. Details of the compensation will be clearly communicated prior to participation. No identifiable images of the participants will be included in the paper or supplementary materials. If such images are necessary, explicit consent will have been obtained from the individuals. Relevant consent forms and written communications will be uploaded during resubmission.

### Sample Size

The sample size (N) was calculated using the standard formula:

N = ((Z_α/2_ + Z_β_)^2^ × [P_1_(1 − P_1_) + P_2_(1 − P_2_)]) / (P_2_ − P_1_)^2^

Z_α/2_ = 1.96 for a 95% CI, representing the chosen level of statistical significance; Z_β_ = 0.84, which corresponds to 80% power; N represents the minimum number of samples required per group; and primary variable = reliability percentage.

Based on [12], the reliability of smartphone for classic shade determination = 7.7%, and the reliability of DSLR camera for classic shade determination = 15.4%.

Using a 5% level of significance and 80% power, the sample size calculation is as follows.

N = (1.96 + 0.84)^2^ × [0.077 (1 − 0.077) + 0.154 (1 − 0.154)] / (0.154 − 0.077)^2^

This yields a minimum required sample size of 221 participants per group.

### Expected Outcome

The primary outcome will be to evaluate if existing digital technologies, that is, DSLR camera and smartphone camera are equally efficient as the intraoral scanner for shade selection. The secondary outcome will be to establish a standardized protocol that ensures accuracy and efficiency of tooth shade selection by using readily available, economical digital devices.

### Statistical Analysis

R software (version 4.3; R Foundation for Statistical Computing) will be used to calculate all the results. Descriptive statistics (mean, standard deviation) will be calculated for tooth shade measurements obtained from each method. Intermethod agreement will be evaluated using Bland-Altman plots or intraclass correlation coefficients. Subgroup analyses will be conducted to explore potential factors influencing intermethod agreement (eg, tooth condition, presence of restorations). Sensitivity and specificity analyses will be performed to evaluate the accuracy of smartphone and DSLR cameras compared to that of intraoral scanners by using the latter as the reference standard. Statistical significance will be set at *P*<.05. Additionally, a paired 2-sided *t* test or Wilcoxon signed-rank test will be used to compare the mean shade differences between each pair of methods (intraoral scanner vs smartphone camera, intraoral scanner vs DSLR camera, smartphone camera vs DSLR camera). The effectiveness of a smartphone and DSLR camera in various dental procedures such as examining the existence of restorations or enamel anomalies will be assessed in a subgroup analysis. In this study, we will test statistical assumptions (eg, normality, homoscedasticity) and apply multiple testing corrections (eg, Bonferroni or false discovery rate) to ensure valid inferences. The intraoral scanner will serve as the reference standard for sensitivity and specificity analyses, using ΔE ≤ 2.0 as the threshold for clinically acceptable shade matches.

## Results

This study was intramurally funded in December 2024. Data collection is scheduled to commence following the publication of this study protocol. As of submission, no participants have been recruited, and data analysis is yet to begin. Results are expected to be completed and published in early January 2026.

## Discussion

A smartphone, DSLR camera, and intraoral scanner will be used to capture and match the shade of a patient's teeth. The smartphone offers a simple portable tool for basic shade reference, while the DSLR camera provides high-quality, controlled images for accurate shade matching under standardized lighting. The intraoral scanner creates a detailed 3D map of the teeth, enabling precise, customized shade selection that can be compared with physical shade guides for optimal restoration outcomes. Advancements in digital dentistry, such as colorimeters, intraoral scanners, and computer-aided shade matching systems, have significantly transformed the shade selection process [13]. These technologies have greatly improved treatment outcomes and patient satisfaction by offering greater accuracy, efficacy, and consistency in shade matching. This study focuses on evaluating the efficiency of smartphone cameras and DSLR cameras in the field of tooth shade selection. Our research seeks to determine their utility as alternatives to subjective methods and cost-intensive objective method for shade selection in dentistry. A study conducted by Yung et al [14] compared the color consistency of digital images captured in a clinical setting by using smartphone cameras and single-lens reflex (SLR) cameras. They found that color accuracy is not considerably increased by using a polarizer. An effective substitute would be a smartphone camera that has an external light source attached to it.

Ziadeh et al [15] assessed the repeatability of the intraoral scanner for selecting dental shades in the anterior and posterior regions. They found that the intraoral scanner demonstrated better repeatability in the anterior region when compared to the visual method, and the difference between the two approaches is clinically acceptable. Faezeh et al [12] examined the validity of color selection techniques by using visual assessments, intraoral scanning, and photography (using an SLR camera and a smartphone). By evaluating and contrasting the dependability of the 3 most popular methods of color selection, that is, visual assessment, intraoral scanning, and photography, oral scanners showed more accurate results.

Abu-Hossin et al [16] compared the use of digital techniques for visual shade selection and evaluated the intraoral scanners' reproducibility. They found that while intraoral scanners are a useful addition for color determination, the visual approach should also be used for confirmation. Moussa et al [17] evaluated the measurements in photos taken with a DSLR, intraoral scanner, and smartphone camera at various distances. According to their study, the measurements of the maxillary right incisors using an intraoral scanner, DSLR, and smartphone (at a minimum distance of 24 cm) did not differ. Both the intraoral scanner and the visual method showed color differences in the central and canine regions that were below the perceptibility threshold—implying that the differences were so small that they could not be noticed by the human eye; these findings are consistent with the investigation performed by Mehl et al [18]. Ebeid et al [19] examined 3 intraoral scanners, namely, CEREC Omnicam (Dentsply Sirona), CEREC Primescan, and 3Shape Trio (Copenhagen, Denmark) by using the Vita Easyshade V spectrophotometer as the control. Ten distinct shades of Vita Mark II blocks were employed in the investigation. Each piece of equipment measured 10 colors per specimen by using the Vitapan Classical shading technique. The accuracy and repeatability data were obtained. According to their study, the accuracy and repeatability of all the intraoral scanners fell short of expectations. Therefore, the authors recommended using the color detection capability of the intraoral scanners with caution. Rutkunas et al [20] compared the accuracy of the intraoral digital 13 scanner, 3Shape Trios, with that of a spectrophotometer, SpectroShade, as a control, and they found that the Trios' mean accuracy for the Vitapan Classical shade tabs was just 27.5%, whereas that for the Vitapan 3D Master shade tabs was 53.3%. Fattouh et al [21], Gasparik et al [22], and Moussaoui et al [23] compared the accuracy, repeatability, and reproducibility of digital intraoral scanners for shade selection and verified whether intraoral optical scanning devices can be used to determine the color of restorations without requiring additional conventional color-measurement methods and concluded that the intraoral scanner Trios color could be an alternative to other methods for shade selection. Czigola et al [24] evaluated the shade measurement function of a digital scanning system (3Shape Trios 3) in relation to visual shade determination (Vita Classical and Vita Linearguide) and a digital spectrophotometry device (Vita Easyshade). Intraoral scanners proved to be as accurate as spectrophotometers; they could serve as objective and time-efficient tools for shade selection and could be seamlessly integrated into comprehensive patient case documentation, as reported by Vitai et al [25]. Although previous research has compared the accuracy of intraoral scanners and smartphone cameras, our study expands the scope by evaluating all 3 digital technologies together by comparing the smartphone camera, DSLR camera, and intraoral scanner to determine how each performs in tooth shade selection. This allows for a more comprehensive assessment of their relative accuracy and clinical usefulness. This study is an attempt to confirm the use of digital technologies for shade selection; if we obtain positive results, further research is warranted to ascertain its universal applicability.

When comparing the reliability of AI-assisted technologies such as intraoral scanners to that of DSLR and smartphone cameras, it is commonly anticipated that intraoral scanners will exhibit higher reliability. Intraoral scanners are specifically designed for dental applications, providing detailed and accurate images of intraoral structures, including teeth. Their direct intraoral capture minimizes the potential sources of error such as variations in lighting or camera positioning, which can affect the reliability of images obtained by DSLR and smartphone cameras. Additionally, intraoral scanners often incorporate advanced technology and software algorithms tailored for precise shade matching and analysis, further enhancing their reliability in dental practice. Therefore, in the context of tooth shade evaluation, it is expected that intraoral scanners will generally offer higher reliability compared to DSLR and smartphone cameras.

## Abbreviations

AI: artificial intelligence
DSLR: digital single-lens reflex
SLR: single-lens reflex

## Data Availability

Data sharing is not applicable to this study as no datasets were generated or analyzed during this study.

## References

[1] Akl MA, Mansour DE, Zheng F (2023). The role of intraoral scanners in the shade matching process: a systematic review. J Prosthodont.

[2] Brandt J, Nelson S, Lauer H, von Hehn U, Brandt S (2017). In vivo study for tooth colour determination—visual versus digital. Clin Oral Invest.

[3] Hardan L, Bourgi R, Cuevas-Suárez Carlos Enrique, Lukomska-Szymanska M, Monjarás-Ávila Ana Josefina, Zarow M, Jakubowicz N, Jorquera G, Ashi T, Mancino D, Kharouf N, Haikel Y (2022). Novel trends in dental color match using different shade selection methods: a systematic review and meta-analysis. Materials (Basel).

[4] Khashayar G, Bain PA, Salari S, Dozic A, Kleverlaan CJ, Feilzer AJ (2014). Perceptibility and acceptability thresholds for colour differences in dentistry. J Dent.

[5] Pimental W, Tiossi R (2014). Comparison between visual and instrumental methods for natural tooth shade matching. Gen Dent.

[6] Özat PB, Tuncel I, Eroğlu E (2013). Repeatability and reliability of human eye in visual shade selection. J Oral Rehabil.

[7] Öngül D, Şermet B, Balkaya MC (2012). Visual and instrumental evaluation of color match ability of 2 shade guides on a ceramic system. J Prosthet Dent.

[8] Berns R (2019). Billmeyer and Saltzman's Principles of Color Technology.

[9] Tabatabaian F, Namdari M, Mahshid M, Vora SR, Mirabbasi S (2024). Accuracy and precision of intraoral scanners for shade matching: a systematic review. J Prosthet Dent.

[10] Raza F, Kumar V, Jacob A, Ali A (2021). Assessment of a smartphone-based software application as a potential digital tool in tooth shade selection: a prospective clinical study. Quintessence Int.

[11] Nayak VM, Sulaya K, Venkatesh SB (2024). Current trends in digital shade matching - A scoping review. Jpn Dent Sci Rev.

[12] Atri F, Memarian M, Rahmani F, Pirooz P Comparison of color selecting methods: reliability, including visual evaluation, intraoral scanning, and photography (SLR camera and smartphone). Research Square.

[13] Derdilopoulou F, Zantner C, Neumann K, Kielbassa A (2007). Evaluation of visual and spectrophotometric shade analyses: a clinical comparison of 3758 teeth. Int J Prosthodont.

[14] Yung D, Tse AK, Hsung RT, Botelho MG, Pow EH, Lam WY (2023). Comparison of the colour accuracy of a single-lens reflex camera and a smartphone camera in a clinical context. J Dent.

[15] Ziadeh CM, Habre P, Nasr L, Haddad H (2023). Dental color matching: a comparison between visual and digital shade selection repeatability in the anterior and posterior region: a clinical study. Current Research in Dentistry.

[16] Abu-Hossin S, Onbasi Y, Berger L, Troll F, Adler W, Wichmann M, Matta R (2023). Comparison of digital and visual tooth shade selection. Clin Exp Dent Res.

[17] Moussa C, Hardan L, Kassis C, Bourgi R, Devoto W, Jorquera G, Panda S, Abou Fadel Roy, Cuevas-Suárez Carlos Enrique, Lukomska-Szymanska M (2021). Accuracy of dental photography: professional vs smartphone's camera. Biomed Res Int.

[18] Mehl A, Bosch G, Fischer C, Ender A (2017). In vivo tooth-color measurement with a new 3D intraoral scanning system in comparison to conventional digital and visual color determination methods. Int J Comput Dent.

[19] Ebeid K, Sabet A, Della Bona A (2021). Accuracy and repeatability of different intraoral scanners on shade determination. J Esthet Restor Dent.

[20] Rutkūnas Vygandas, Dirsė Julius, Bilius Vytautas (2020). Accuracy of an intraoral digital scanner in tooth color determination. J Prosthet Dent.

[21] Fattouh M, Kenawi LM, Aboelela OA (2021). Repeatability of visual, spectrophotometer and intraoral scanner methods in shade matching: a comparative in-vivo study. Int J Dent Oral Sci.

[22] Gasparik C, Grecu AG, Culic B, Badea ME, Dudea D (2015). Shade-matching performance using a new light-correcting device. J Esthet Restor Dent.

[23] Moussaoui H, El Mdaghri M, Gouma A, et al (2018). Accuracy, repeatability and reproducibility of digital intraoral scanner for shade selection: current status of the literature. Oral Health Dental Sci.

[24] Czigola A, Róth Ivett, Vitai V, Fehér Dóra, Hermann P, Borbély Judit (2021). Comparing the effectiveness of shade measurement by intraoral scanner, digital spectrophotometer, and visual shade assessment. J Esthet Restor Dent.

[25] Vitai V, Németh Anna, Teutsch B, Kelemen K, Fazekas A, Hegyi P, Németh Orsolya, Kerémi Beáta, Borbély Judit (2025). Color comparison between intraoral scanner and spectrophotometer shade matching: a systematic review and meta-analysis. J Esthet Restor Dent.

